# Microembolizations in the arterial cerebral circulation during atrial fibrillation ablation: cryoballoon technique advantages and neurocognitive safety—results of a prospective observational study

**DOI:** 10.1093/europace/euae222

**Published:** 2024-08-24

**Authors:** Damir Erkapic, Konstantinos Roussopoulos, Marko Aleksic, Kay Felix Weipert, Korkut Sözener, Karel Kostev, Jens Allendörfer, Josef Rosenbauer, Samuel Sossalla, Dursun Gündüz, Christian Tanislav

**Affiliations:** Diakonie Klinikum Jung Stilling, Medical Clinic II, Department of Cardiology, Rhythmology and Angiology, Wichernstraße 40, 57074 Siegen, Germany; University Clinic of Giessen, Medical Clinic I, Department of Cardiology and Angiology, Klinikstraße 33, 35392 Giessen, Germany; Diakonie Klinikum Jung Stilling, Medical Clinic II, Department of Cardiology, Rhythmology and Angiology, Wichernstraße 40, 57074 Siegen, Germany; Diakonie Klinikum Jung Stilling, Medical Clinic II, Department of Cardiology, Rhythmology and Angiology, Wichernstraße 40, 57074 Siegen, Germany; Diakonie Klinikum Jung Stilling, Medical Clinic II, Department of Cardiology, Rhythmology and Angiology, Wichernstraße 40, 57074 Siegen, Germany; Diakonie Klinikum Jung Stilling, Medical Clinic II, Department of Cardiology, Rhythmology and Angiology, Wichernstraße 40, 57074 Siegen, Germany; Klinikum Hanau, Department of Rhythmology, Leimenstraße 20, 63450 Hanau, Germany; Philipps-University Marburg, Department of Epidemiology, Baldingerstraße, 35037 Marburg, Germany; Neurological Clinic Bad Salzhausen, Am Hasenprung 6, 63667 Nidda, Germany; Diakonie Klinikum Jung Stilling, Medical Clinic II, Department of Cardiology, Rhythmology and Angiology, Wichernstraße 40, 57074 Siegen, Germany; University Clinic of Giessen, Medical Clinic I, Department of Cardiology and Angiology, Klinikstraße 33, 35392 Giessen, Germany; Diakonie Klinikum Jung Stilling, Medical Clinic II, Department of Cardiology, Rhythmology and Angiology, Wichernstraße 40, 57074 Siegen, Germany; University Clinic of Giessen, Medical Clinic I, Department of Cardiology and Angiology, Klinikstraße 33, 35392 Giessen, Germany; Diakonie Klinikum Jung Stilling, Department of Geriatrics and Neurology, Wichernstrasse 40, 57074 Siegen, Germany

**Keywords:** Microembolization, MES, Neurocognitive safety, PVI, Cryoballoon, LARA

## Abstract

**Aims:**

The significance of micro-embolic signals (MESs) during atrial fibrillation (AF) ablation is unclear. Previous studies had limitations, and cryoballoon (CB) ablation patients were under-represented. Minimizing MESs is recommended due to their uncertain neurocognitive impact.

**Methods and results:**

This prospective observational study included AF patients from a German centre between February 2021 and August 2022. Patients were equally divided into paroxysmal (Group A) and persistent (Group B) AF. Group A received cryoballoon-pulmonary vein isolation only, while Group B also had left atrial roof ablation. MESs were detected using transcranial Doppler ultrasonography during ablation. Neurocognitive status was assessed pre- and post-procedure and at 3 months using the CERAD Plus battery. The study analyzed 100 patients with a median age of 65.5 years. A total of 19 698 MESs were observed, with 80% being gaseous and 20% solid in origin, primarily occurring during pulmonary vein angiography and the balloon freeze and thawing phase. The median MES per patient was 130 (IQR: 92–256) in total, 298 (IQR: 177–413) in bilateral (36%), and 110 (IQR: 71–130) in unilateral (64%) recordings. No significant difference in total MES counts was found between the groups. None of the 11 neuropsychological tests showed cognitive decline post-procedure or at 3 months.

**Conclusion:**

Our observations confirm that neurocognitive abilities are not affected either 24 h or 3 months after AF ablation using the CB technique. However, despite the low MES burden associated with the CB, more work is needed to reduce small embolic events during AF ablation.

## Graphical Abstract

**Graphical abstract:**
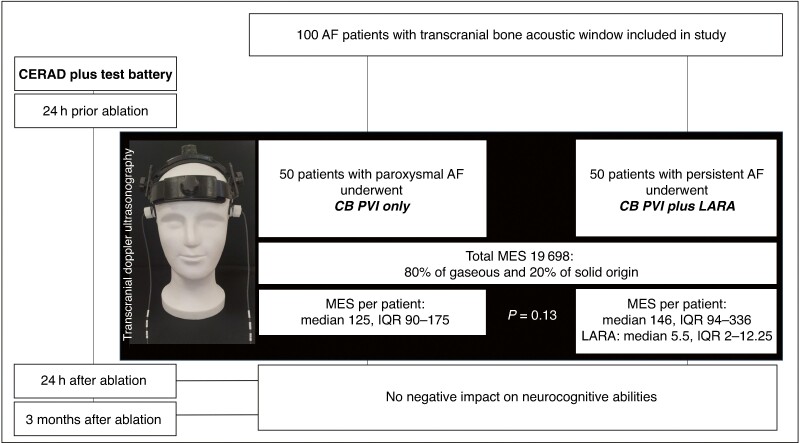

**What’s new?**

- Largest prospective observational cohort study to date on transcranial Doppler ultrasonography-detected microembolizations during atrial fibrillation (AF) ablation.
- Observed low microembolization burden in the cerebral arterial circulation during AF ablation in paroxysmal and persistent AF patients using cryoballoon technique.
- First evaluation of the microembolization impact of an additional left atrial roof ablation (LARA).
- First evaluation of operator dependency on the microembolic burden.
- Step-by-step demasking of microembolizations during cryoballoon ablation, showing 80% of gaseous and 20% of solid origin.
- Comprehensive 11 neuropsychological subtests, showing no negative impact on cerebral function 24 h and 3 months after atrial fibrillation ablation and beyond using cryoballoon technique.

## Introduction

During catheter-based ablation for atrial fibrillation (AF), both gaseous and solid emboli form in the heart, which have been shown to embolize into the cerebral arterial circulation.^1^ The clinical significance of these so-called microembolizations has not yet been clearly determined.^2^ While most of these microembolizations appear to be clinically silent, some authors describe temporary neurocognitive impairments in AF patients immediately after ablation treatment.^2–4^ Since the use of AF ablation is growing steadily worldwide thanks to its effective rhythm control, and cryoballoon (CB) ablation seems to be increasingly establishing itself as the first-line therapy, further investigation into the safety profile of this procedure is warranted.^5–7^ All studies published to date that have examined the frequency and type of microembolizations and their neurocognitive outcome after AF ablation have included heterogeneous patient populations with mostly small sample sizes and different ablation energy sources.^3,4,8–10^ In view of these circumstances, we conducted the largest prospective study on this topic to date, aiming to identify the cerebral embolic activity during AF ablation exclusively using the CB technique and assessing its impact on neurocognitive outcomes.

## Methods

### Study design and study population

The detailed protocol and methodology of this study as well patients’ baseline and procedural characteristics have been published previously.^11^

In brief, the study was designed as a prospective observational study of AF patients treated with the CB ablation technique. The study protocol was approved by the Ethics Committee of the University of Münster, Germany (AZ 2019-779-f-S), and patients provided informed consent. The study included patients with paroxysmal or persistent atrial fibrillation, defined on the basis of the current guidelines,^12^ who met specified inclusion criteria. This primarily involved the presence of a transcranial acoustic bone window for the detection of microembolic signals (MESs) using ultrasound technology. Patients were excluded from the study if pre-defined relative contraindications for pulmonary vein isolation (PVI) were present or if medical conditions existed which meant that (1) a low likelihood of success of the ablation treatment was expected, or (2) the neurocognitive abilities of the patients could have been affected.

Patients were divided into two groups based on the type of AF they had: Group A with paroxysmal AF receiving PVI only, and Group B with persistent AF receiving PVI and additional left atrial roof ablation (LARA). The main objective of the study was to investigate the burden of MES during the procedure and their potential impact on cognitive ability. The study design involved a cross-sectional analysis of MES and a longitudinal cohort study to assess cognitive decline. We also aimed to determine whether there was a correlation between cognitive decline and the burden of MES.

### MES detection and interpretation

**Figure 1:**
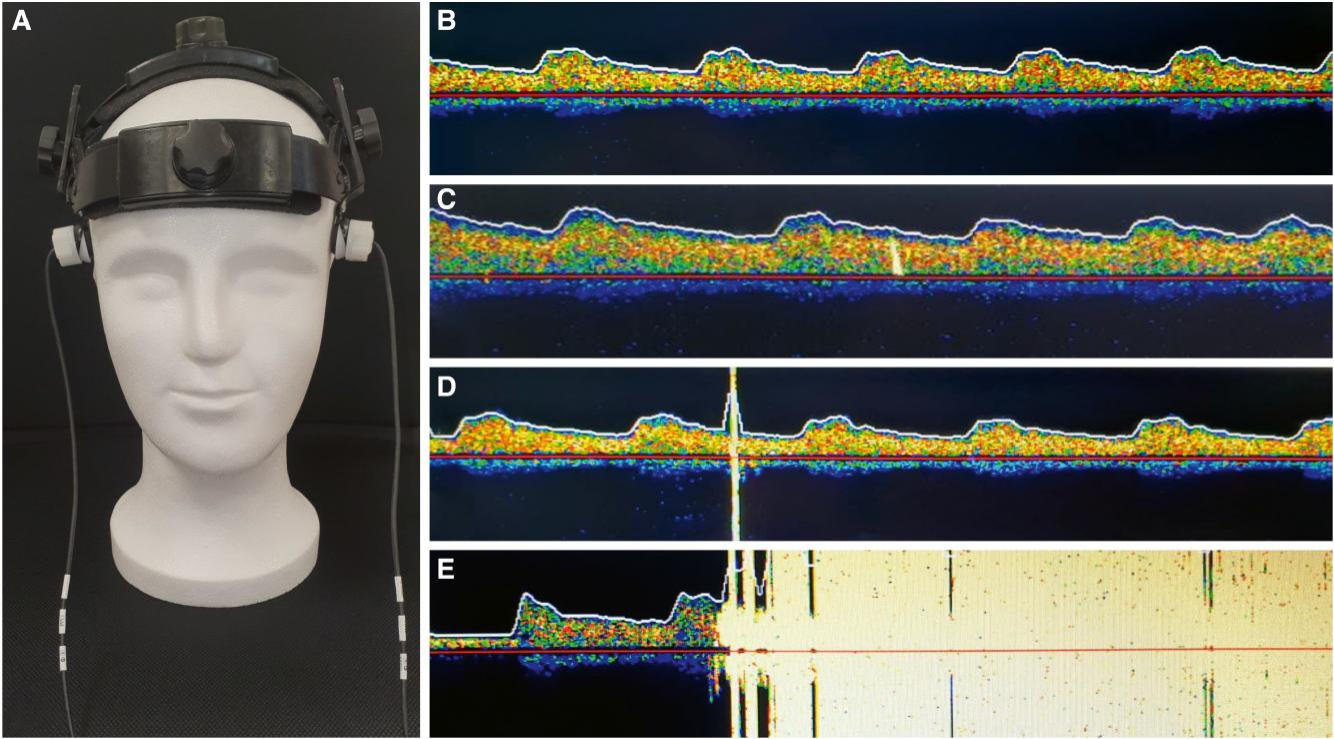
Microembolic signal detection using Transcranial Doppler Ultrasonography. (*A*) Transcranial Doppler ultrasonography displaying the flow signal over the arteria cerebri media; (*B*) normal flow profile over the middle cerebral artery; (*C*) high-intensity signal (HITS) with strictly unidirectional signal within the Doppler spectrum implying a ‘solid’ embolus; (*D*) large, high-intensity bidirectional signal (HITS) exceeding the Doppler spectrum implying a ‘gaseous’ embolus; (*E*) many (innumerable) HITS, indicating a shower. *Adapted and modified from Erkapic et al.*^11^ HITS, high-intensity signal.

**Figure 2:**
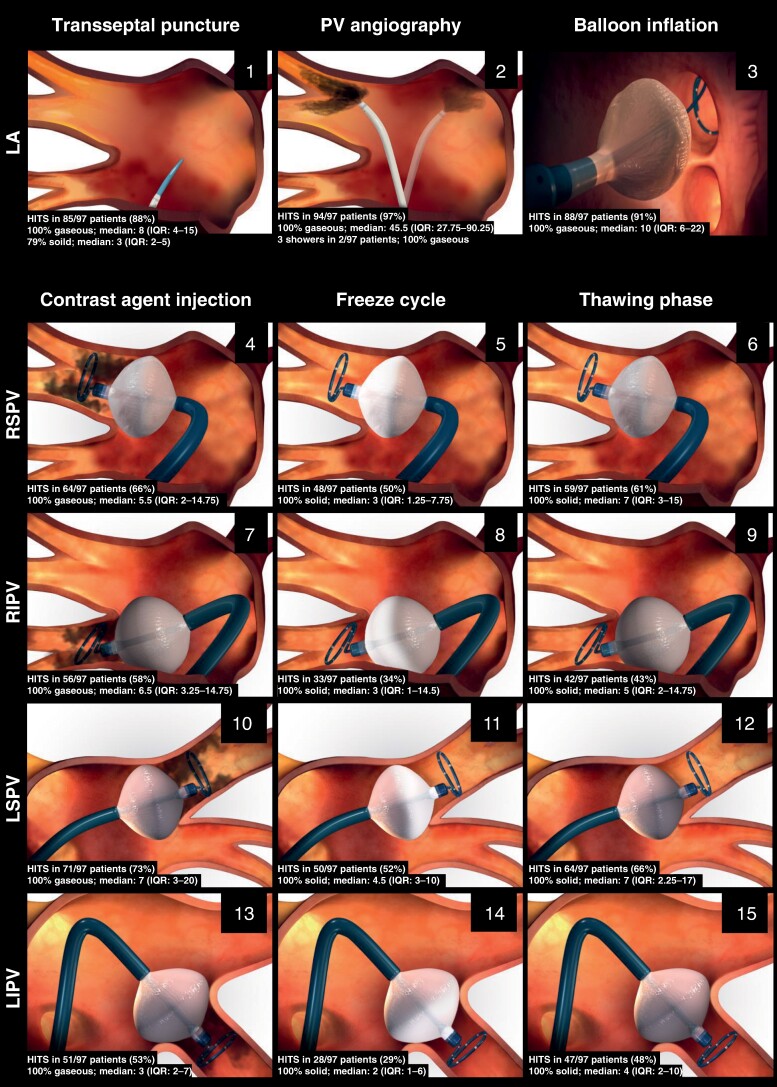
Timeline step-by-step demasking of number and type of microembolic signals during cryoballoon pulmonary vein isolation. HITS, high-intensity signal; LA, left atrium; LIPV, left inferior pulmonary vein; LSPV, left superior pulmonary vein; PV, pulmonary vein; RSPV, right superior pulmonary vein; RIPV, right inferior pulmonary vein.

Transcranial Doppler (TCD) ultrasonography is an established method in neurology that enables the real-time detection of microemboli, which may form during procedures such as left atrial ablation and subsequently embolize into the brain. MES were continuously recorded using TCD during the ablation procedures (*Figure 1A*). Using TCD emboli can be detected through their audible and visible impedance jumps within the derived frequency spectrum of the middle cerebral artery. This is possible due to the strong reflection of ultrasound waves at the interface between the embolus and the surrounding blood.^13^ Several studies have confirmed that signals from TCD are caused by ‘gaseous’ or ‘solid’ emboli, measuring approximately 5 μm (gaseous) to 100 μm (solid).^14,15^ In our study, non-invasive TCD was performed using the DWL Multi-Dop T (DWL Elektronische Systeme GmbH, Sipplingen, Germany) to visualize the flow signal over the middle cerebral artery (*Figure 1B*). The methodological aspects of MES detection using TCD ultrasound adhered to internationally accepted recommendations.^16^ Briefly, the transcranial transducer was fixed to the temporal bone immediately before the procedure began, after locating the optimal acoustic window—ideally with bilateral derivation of the middle cerebral artery Doppler spectrum. The sample gate was set to 8 mm, and the Doppler gain was reduced. Signal recording commenced just before the inguinal puncture and continued until the inguinal sheaths were removed. MES detection distinguished between (1) single MES or HITS (High-Intensity Transient Signal) with acoustic and visual uni- or bidirectional signals and an impedance >8 dB above the baseline (*Figure 1C, D*) and (2) showers, defined as HITSs that could not be individually counted, indicating pronounced embolizations (*Figure 1E*). By definition, ‘solid’ emboli were strictly unidirectional within the Doppler spectrum and had an acoustic impedance > 8 dB above the baseline (*Figure 1C*). ‘Gaseous’ emboli were large, high-intensity bidirectional signals exceeding the Doppler spectrum (*Figure 1D*). Since the size and composition of an embolus independently influence the intensity of an MES, it can be challenging to classify a detected MES as distinctly ‘gaseous’ or ‘solid.’ Therefore, another parameter was included in our study to enhance the accuracy of identifying and classifying the embolus as gaseous or solid: the timing of MES occurrence. This was tracked using a chronological log of each procedural step. The clock of the DWL Multi-Dop T device was synchronized with the clock of the electrophysiological registration unit (BARD, Boston Scientific, Marlborough, MA, USA). To avoid influencing the electrophysiologists and the assisting team directly involved in the ablation procedure, the acoustic signals for MES detection were muted, and the screen displaying the MES was not visible to the ablation team. If the detection signal was lost during the continuous recording of the flow signal over the middle cerebral artery due to slippage of the ultrasound probe on the patient’s temple, repositioning was immediately performed by a physician not involved in the ablation procedure.

At the end of the procedure, all HITSs and showers were manually counted and assigned to the various steps of the ablation procedure based on the time log and categorized as either ‘gaseous’ or ‘solid’ emboli. Additionally, to interpret the potential origin of the solid MES, the following approach was taken: if a ‘solid’ MES was detected during the freeze cycle or the thawing phase, it was attributed to an ice particle. Conversely, if a ‘solid’ MES was detected independently of the CB thawing phase or freeze time, its origin was most likely attributed to a cardiac tissue fragment. The evaluation was conducted by two neurologists experienced in TCD technique and cerebral MES detection, each blinded to the other’s work. Each neurologist collaborated with an electrophysiologist familiar with the CB ablation technique. In cases of disagreement, a consensus read involving all four medical colleagues was conducted. After discussion, a consensus judgment was documented and considered for further analysis.

### CB-assisted ablation procedures and peri-procedural characteristics

All ablation procedures were conducted using a biplane fluoroscopy system (Siemens Artis Zee biplane, Erlangen, Germany) with RAO 30° and LAO 60° views. Following a transseptal puncture using an SL-1 sheath and a BRK-1 needle (Abbott, St. Paul, MN, USA), angiography of the left atrial and pulmonary veins was performed. Rapid pacing with a cycle length of 300 ms was applied through a decapolar catheter (ViaCath™, Biotronik, Berlin, Germany) in the right ventricle immediately prior to angiography. Subsequently, 15 mL of contrast agent was manually injected into the left superior pulmonary vein (LSPV), followed by the right superior pulmonary vein (RSPV) via the SL-1 sheath. The contrasted borders of the left atrium, pulmonary veins, and transseptal puncture site were then traced on the examiner’s monitor using a board marker to ensure safe movement and positioning of the CB during the ablation procedure.

All ablations were exclusively performed using the 4th generation CB Arctic Front Advance Pro 28 mm (Medtronic Inc., Mounds View, MN, USA) following the replacement of the SL-1 sheath with the CryoAdvance sheath (Medtronic Inc., Mounds View, MN, USA) utilizing the Seldinger technique. Pulmonary vein signals were mapped with an inner lumen spiral mapping catheter (Achieve™ catheter, 20 mm diameter, Medtronic) before, during, and after each cryoenergy application. Guided by the Achieve™ catheter, the 28 mm CB was advanced through the sheath into the left atrium, inflated proximally to the pulmonary vein ostium, and then gently pushed to occlude the pulmonary vein. Vessel occlusion and atrial regurgitation were assessed by selectively injecting contrast medium via an automatic injection pump (CVi™, ACIST Europe B.V.) at a standardized volume of 5 mL, flow rate of 3 mL/s, and pressure of 350 psi. After achieving optimal occlusion, a 240-s freeze–thaw cycle was performed. If a temperature of −60°C was reached before the completion of the 240-s freeze–thaw cycle, the freeze–thaw time was terminated earlier. During ablation of the septal pulmonary veins, the decapolar catheter was positioned in the superior vena cava for ipsilateral phrenic nerve stimulation. Phrenic nerve function was monitored using diaphragmatic compound motor action potentials (CMAPs). Ablation was immediately halted if substantial decreases in CMAPs were observed, indicating weakening or loss of diaphragmatic contraction.

Successful isolation of the pulmonary veins was defined by demonstrating the elimination of pulmonary vein potentials measured at the ostium of each pulmonary vein using a 20 mm Achieve™ catheter (Medtronic), as well as demonstrating an entry and exit block via the Achieve™ catheter and the decapolar CS-catheter (ViaCath™, Biotronik, Berlin, Germany). Additional LARA was conducted in all patients with persistent AF. Sequential, overlapping 180-s freezes were applied along the left atrial roof, starting near the position used for left superior PVI. This procedure involved gentle clockwise rotation combined with gentle sheath retraction and incremental advancement of the CB until the position used for right superior PVI was reached. The Achieve™ catheter was anchored in the left superior pulmonary vein for all CB positions at the left atrial roof.

In cases of ongoing intraprocedural AF, electrical cardioversion (200 Joules biphasic AP) was performed, followed by verification of PVI and a conduction block at the left atrial roof, as previously described.^17,18^ In cases where additional typical atrial flutter was present, the cavotricuspid isthmus (CTI) was ablated using an 8 mm gold-tip non-irrigated radiofrequency catheter (AlCath Flutter LT G, Biotronik, Berlin, Germany) with power settings of 60W/60°C. All CTI ablations were conducted after left atrial ablation and retraction of the transseptal sheath into the right atrium.

All ablations in the study were performed by three electrophysiologists with varying levels of experience in CB ablation: Investigator 1 had conducted between 50 and 100 CB ablations, Investigator 2 had performed over 100 but fewer than 500 CB ablations and Investigator 3 had carried out over 500 CB ablations. Whenever possible, the number of ablation procedures completed was distributed equally among the three electrophysiologists.

The intake of direct oral anticoagulants (DOACs) was paused only on the morning of the ablation procedure. DOAC intake was resumed at adequate doses two hours after the procedure, provided that pericardial effusion had been ruled out and the inguinal puncture site was unremarkable. For patients on phenprocoumon, the procedure was performed with an international normalized ratio (INR) of 2.0–3.0. Transoesophageal echocardiography was performed if oral anticoagulant intake had been insufficient within the four weeks before the procedure and in all cases of persistent atrial fibrillation. During the procedure, unfractionated heparin (Panpharma, Trittau, Germany) was administered intravenously to achieve a target activated clotting time (ACT) of ≥300 s. A standardized body weight and INR-adapted dosage, as described by Hamam et al., was used for this purpose.^19^ Although this formula was originally developed for use with vitamin K antagonists, it has also proven effective in our clinical practice with DOACs, administering heparin at a dosage of 184 units/kg. Of this, the first 5 000 units were administered after the groin puncture, and the remaining units were given after the transseptal puncture. If the target ACT of greater than or equal to 300 s was not achieved in the subsequent standardized 20-min control ACT measurement, further heparin bolus doses were given at the discretion of the investigator. ACT values were measured with the ACT Plus^TM^ (Medtronic, Minneapolis, MN) every 20 min. No protamine was administered at the end of the procedure. After the removal of the catheters and venous sheaths, the access site was closed with subcutaneous temporary purse-string sutures.^20^

### Neuropsychological examinations

Neuropsychological examinations were performed before 24 h prior the ablation procedure, within 24 h after the procedure, and at a follow-up appointment three months later. The examinations assessed comprehensive cognitive functions using the CERAD Plus battery, which consists of 11 neuropsychological tests and covers domains such as executive, memory, language, and visuospatial skill.^11^ All examinations were carried out by two physicians trained for this purpose in accordance with the known specifications.^21^

## Statistical analysis

Categorical variables were reported as frequencies and percentages. Normal distribution was assessed using the Kolmogorov-Smirnov one-sample test. Nonparametric data were analyzed using a two-tailed Mann–Whitney *U*-test. The Yates χ^2^ test was utilized to compare relative frequencies. Statistical analyses were conducted using SPPS software (version 22.0. IBM Corporation, Armonk, NY, USA).

## Results

The baseline and peri-procedural ablation characteristics are displayed in *Tables 1* and *2*, respectively. Due to insufficient TCD signal quality during the procedure, three patients—two from Group A and one from Group B—had to be excluded from the analysis retrospectively. MESs were detected in all patients. Bilateral recording of MES was possible in 35 (36%) of the 97 patients investigated, while 28 patients (29%) exhibited unilateral signal recording over the right middle cerebral artery (RMCA), and 34 (35%) over the left middle cerebral artery (LMCA). No significant differences in the clinical characteristics of the patients with and without bilateral recordings could be observed (*Table 3*). The distribution of uni- and bi-lateral recordings among patients with paroxysmal AF and persistent AF showed no significant differences (*P* = 0.068), *Table 3*. For the entire group, 19 698 MES were counted. In four patients there was judgment difference in the MES count by the blinded neurologists, so both repeated it in consensus read. Therefore an inter-rater agreement on 93/97 (95.9%) was obtained. Overall, a median of 130 MES (IQR: 92–256) was recorded in each patient. With respect to the available transtemporal acoustic bone windows, the following MES were counted per patient in median: 298 (IQR: 177–413) bilateral and 110 (IQR: 71–130) unilateral. The distribution of unilateral MES counts (LMCA and RMCA) is displayed in *Table 4*. There was no significant difference in the total number of MES between Group A (*n* = 48) with paroxysmal AF and B (*n* = 49) with persistent AF (median 125, IQR: 90–175 vs. median 146, IQR: 94–336; *P* = 0.13).

**Table 1:** Baseline characteristics

Baseline characteristics				
	Total (*n* = 100)	Paroxysmal AF (*n* = 50)	Persistent AF (*n* = 50)	*P*
**Age** (median, IQR^a^) (years)	65.5 (58.2–72.4)	66.1 (57.3–71.4)	65.5 (58.8–74.4)	0.564
**Sex**				
Male	69 (69%)	28 (56%)	41 (82%)	**0**.**009**
Female	31 (31%)	22 (44%)	9 (18%)	
**BMI** (median, IQR^a^), (kg/m^2^)	28.5 (25.5–33.4)	27.4 (23.9–31.8)	30.3 (26.7–34.7)	**0**.**016**
**CHA2DS2VASc** (median, IQR^a^, range)	2 (1–3, 0–7)	2 (1–3)	2 (1–4)	0.056
**Echocardiography**				
Left atrium index (mL/m^2^) (median, IQR^a^)	39.0 (29.9–50.9)	31.7 (24.3–38.9)	48.1 (38.5–56.1)	**<0**.**001**
Mitral insufficiency I–II° (median, IQR^a^, range) (*n* = 77)	I° (I°–I°; I°–II°)	I° (0–I°)	I° (I°–I°)	**0**.**037**
Left ejection fraction (median, IQR^a^, range) (%)	60 (55–60; 40–75)	60 (60–65)	55 (50–60)	**<0**.**001**
**Co-morbidities**				
Hypertension	71 (71%)	33 (66%)	38 (76%)	0.387
Diabetes mellitus	18 (18%)	7 (14%)	11 (22%)	0.378
Coronary artery disease	21 (21%)	10 (20%)	11 (22%)	0.999
Sleep apnoea	11 (11%)	3 (6%)	8 (16%)	0.201
Heart insufficiency				
NT-Pro-BNP (ng/L) (median, IQR) (*n* = 61)	417.0 (86.5–153.5)	232.5 (138.3–470.5)	742.0 (206.5–2178.5)	**0**.**004**
Kidney disease				
eGFR (mL/min/1.73m^2^) (median, IQR)	76.0 (64.4–88.9)	77.5 (66.8–89.1)	75.2 (58.4–89.0)	0.295
Gastroesophageal reflux disease	5 (5%)	3 (6%)	2 (4%)	0.999
Previous stroke	8 (8%)	2 (4%)	6 (12%)	0.269
**Intake of oral anticoagulants prior ablation**	95 (95%)	45 (90%)	50 (100%)	0.056
Apixaban 5 mg twice/day	65 (65%)	30 (60%)	35 (70%)	0.402
Apixaban 2.5 mg twice/day	4 (4%)	1 (2%)	3 (6%)	0.617
Rivaroxaban 20 mg once/day	6 (6%)	4 (8%)	2 (4%)	0.678
Rivaroxaban 15 mg once/day	0 (0%)	0 (0%)	0 (0%)	>0.999
Edoxaban 60 mg once/day	11 (11%)	6 (12%)	5 (10%)	>0.999
Edoxaban 30 mg once/day	3 (3%)	2 (4%)	1 (2%)	>0.999
Dabigatran 150 mg twice/day	0 (0%)	0 (0%)	0 (0%)	>0.999
Dabigatran 110 mg twice/day	0 (0%)	0 (0%)	0 (0%)	>0.999
Phenprocoumon (INR 2–3)	6 (6%)	2 (4%)	4 (8%)	0.678
**Intake of platelet inhibitors**	7 (7%)	1 (2%)	6 (12%)	0.122
Clopidogrel 75 mg once/day	3 (3%)	0 (0%)	3 (6%)	0.242
Aspirin 100 mg once/day	3 (3%)	0 (0%)	3 (6%)	0.242
Prasugrel 10 mg once/day	1 (1%)	1 (2%)	0 (0%)	>0.999

*Adapted and modified from Erkapic et al.* ^11^

AF, atrial fibrillation; BMI, body mass index; eGFR, estimated glomerular Filtration Rate; IQR, interquartile range.

^a^Q1/Q3.

**Table 2:** Peri-procedural ablation characteristics

Peri-procedural ablation characteristics				
	Total (*n* = 100)	Paroxysmal AF (*n* = 50)	Persistent AF (*n* = 50)	*P*
**Heparin dosage** (units) per patient (median, IQR^a^)	18 000 (15 000–21 000)	16 000 (13 500–19 000)	20 000 (16 125–22 000)	**<0**.**001**
**Activated clotting time** (sec) per patient (median, IQR^a^)	328 (320–355)	328 (320–356)	329 (320–345)	0.855
**Pulmonary vein isolation**				
Acute ablation success	100 (100%)	50 (100%)	50 (100%)	0.999
Total number of freezes (median, IQR^a^)	5 (4–5)	5 (4–5)	5 (4–6)	0.172
Cryoenergy application total time (min) (median, IQR^a^)	16.2 (15.1–20.0)	16.0 (15.2–19.5)	17.1 (15.0–22.1)	0.435
RSPV Cryoenergy application time (min) (median, IQR^a^)	4 (3–4)	4 (3–4)	4 (3–5)	0.811
RIPV Cryoenergy application time (min) (median, IQR^a^)	4 (4–4)	4 (4–5)	4 (4–4)	0.698
LSPV Cryoenergy application time (min) (median, IQR^a^)	4 (4–4)	4 (4–4)	4 (4–4)	0.944
LIPV Cryoenergy application time (min) (median, IQR^a^)	4 (4–4)	4 (4–4)	4 (4–4)	0.347
Nadir temperature (°C) (median, IQR^a^)				
Nadir temperature RSPV (°C) (median, IQR^a^)	−53 (−56 to −49)	−54 (−56 to −51)	−52 (−56 to −48)	0.326
Nadir temperature RIPV (°C) (median, IQR^a^)	−51 (−55 to −47)	−52 (−55 to −47)	−50 (−54 to −47)	0.323
Nadir temperature LSPV (°C) (median, IQR^a^)	−48 (−53 to −45)	−48 (−54 to −44)	−49 (−53 to −46)	0.218
Nadir temperature LIPV (°C) (median, IQR^a^)	−46 (−51 to −45)	−46 (−48 to −45)	−48 (−54 to −45)	0.062
**Left Atrial Roof Ablation (LARA)**				
Acute ablation success			41 (82%)	
Total number of freezes (median, IQR^a^, range)			4 (3–4; 3–6)	
Cryoenergy application time (min) (median, IQR^a^)			9 (9–18)	
Nadir temperature (°C) (median, IQR^a^)			−40 (−33 to −46)	
**Additional right isthmus RF-ablation**	11 (11%)	7 (14%)	4 (8%)	0.525

*Adapted and modified from Erkapic et al.* ^11^

IQR, interquartile range; LSPV, left superior pulmonary vein; LIPV, left inferior pulmonary vein; min, minutes; RSPV, right superior pulmonary vein; RIPV, right inferior pulmonary vein; sec, seconds.

^a^Q1/Q3.

**Table 3:** Comparing clinical characteristics by availability of TCD

	Transtemporal acoustic bone window		
	bilateral (*n* = 35)	unilateral (*n* = 62)	*P*
**Age** (median, IQR^a^) (years)	64.0 (58.0–74.0)	68.5 (56.8–72.0)	0.994
**Sex**			
Male (*n* = 66)	25 (71%)	41 (66%)	0.591
Female (*n* = 31)	10 (29%)	21 (34%)	
**BMI** (median, IQR^a^), (Kg/m^2^)	27.3 (25.1–31.2)	29 (25.9–35.1)	0.102
**Co-morbidities**			
Hypertension (*n* = 69)	24 (69%)	45 (73%)	0.816
Diabetes mellitus (*n* = 18)	6 (17%)	12 (19%)	0.788
Previous stroke (*n* = 8)	1 (3%)	7 (11%)	0.147
**CHA2DS2VASc** (median, IQR^a^, range)	2 (1–3)	2 (1–4)	0.229
**Atrial fibrillation**			
paroxysmal (*n* = 48)	13 (37%)	35 (57%)	0.068
persistent (*n* = 49)	22 (63%)	27 (44%)	

BMI, body mass index; IQR, interquartile range.

^a^Q1/Q3.

**Table 4:** MES-burden corresponding to bilateral and unilateral acoustic bone windows

	Total patients (*n* = 97)	MES-burden		
		*Range*	*Median*	*IQR* ^a^
**Transtemporal acoustic bone window**				
Bilateral	35 (36%)	(53–1 000)	298	(177.0–413.0)
Unilateral (total)	62 (64%)	(17–1 000)	110	(71.3–130.0)
Unilateral (LMCA)	34 (35%)	(17–1 000)	113	(71.3–130.0)
Unilateral (RMCA)	28 (29%)	(38–298)	107	(69.0–130.8)

IQR, interquartile range; LMCA, left middle cerebral artery; MES, microembolic signal; RMCA, right middle cerebral artery.

^a^Q1/Q3.

Of 19 698 MES, 15 745 (80%) were counted as gaseous and 3 953 (20%) as solid MES. MES in the form of gaseous HITS (median 22.5; IQR: 13–38.75) were already detected in four patients after venous access but before transseptal puncture. During follow-up, transoesophageal echocardiography confirmed the presence of persistent foramen ovale with an atrial septal aneurysm in all four patients. Transseptal puncture was associated with both gaseous and solid MES, while PV angiography, balloon inflation into the left atrium, and contrast agent injection into each PV after balloon occlusion only showed MES of gaseous origin. On the other hand, solid microembolizations were observed during the freeze cycle and thawing phase. A timeline illustrating the number and type of microemboli during the different procedural steps of CB ablation (*Figure 2*) includes the following steps (1–15): (1) transseptal puncture: median of 8 (IQR: 4–15) gaseous HITS and 3 (IQR: 2–5) solid HITS; (2) PV angiography: 45.5 (IQR: 27.75–90.25) gaseous HITS, with three showers in two patients; all HITS/showers were exclusively of gaseous origin; (3) balloon inflation into the left atrium: 10 (IQR: 6–22) gaseous HITS; (4) RSPV occlusion and contrast agent injection: 5.5 (IQR: 2–14.75) gaseous HITS; (5) freeze cycle at RSPV: 3 (IQR: 1.25–7.75) solid HITS; (6) balloon thawing phase at RSPV: 7 (IQR: 3–15) solid HITS; (7) RIPV occlusion and contrast agent injection: 6.5 (IQR: 3.25–14.75) gaseous HITS; (8) freeze cycle at RIPV: 3 (IQR: 1–14.5) solid HITS; (9) balloon thawing phase at RIPV: 5 (IQR: 2–14.75) solid HITS; (10) LSPV occlusion and contrast agent injection: 7 (IQR: 3–20) gaseous HITS; (11) freeze cycle at LSPV: 4.5 (IQR: 3–10) solid HITS; (12) balloon thawing phase at LSPV: 7 (IQR: 2.25–17) solid HITS; (13) LIPV occlusion and contrast agent injection: 3 (IQR: 2–7) gaseous HITS; (14) freeze cycle at LIPV: 2 (IQR: 1–6) solid HITS; and (15) balloon thawing phase at LIPV: 4 (IQR: 2–10) solid HITS.

Additional performed CTI ablations (*n* = 11) were not associated with the occurrence of MES. Furthermore, there was no correlation between the lowest temperatures during the CB ablation and the type of MES.

Among MES with solid origin, 3 687 (93%) MES occurred during the freeze cycle and thawing phase and 266 (7%) during transseptal puncture.

The introduction of the new haemostatic valve of the Cryoadvance sheath (used in *n* = 77) by Medtronic in April 2021, was not found to have a significant impact on the frequency of MES (median 131, IQR: 105.5–231.0) compared with the 20 patients included in this study with the old haemostatic valve (median 122, IQR: 68.25–363.0), *P* = 0.56.

LARA was associated with fewer MES (28/49 patients; median 5.5, IQR: 2–12.25), all of which were of solid origin and occurred in the thawing phase. Pronounced embolizations, characterized as showers, were observed three times in a total of two patients. These occurred during PV angiography through the transseptal sheath and involved Investigators 1 and 2. The distribution of unilateral and bilateral acoustic bone windows among the three investigators showed no significant difference: unilateral [*n* = 23 (69.7%) vs. *n* = 19 (55.9%) vs. *n* = 20 (66.7%)], bilateral [*n* = 10 (30.3%) vs. *n* = 15 (44.1%) vs. *n* = 10 (33.3%)], *P* = 0.460. While in the unilateral recordings, the lowest MES burden was counted by the most experienced investigator (median 92, IQR: 70–112 vs. median 110, IQR: 59–130 vs. median 125, IQR: 104–148, *P* = 0.041), no significant difference was observed in the bilateral recordings (median 294, IQR: 195–418 vs. median 363, IQR: 152–500 vs. median 247, IQR: 175–375), *P* = 0.637.

Peri-procedural external electrical cardioversion (eCV) was performed in 35 patients (9 in Group A and 26 in Group B) with a biphasic anteroposterior energy of 200 J. ECV was not associated with the occurrence of MES in any patient.

The results of neuropsychological testing are displayed in *Tables 5* and *6*. None of the eleven tests performed revealed any noticeable reduction in cognitive ability, either shortly after the procedure, or even 3 months later. Remarkably, no cognitive decline could be observed even in patients with confirmed pronounced embolizations. A statistically significant improvement in neurocognitive abilities was observed in 5 of the 11 examined domains during the follow-up period. The comparison between patients with persistent vs. paroxysmal AF revealed no differences regarding the CERAD-Plus testing in all three study visits (*Table 6*). Additional performed CTI ablations showed no impact on cognitive function.

**Table 5:** Comparison in neuropsychological testing (CERAD plus battery) 24 h prior to, 24 h after, and 3 months after the ablation procedure

	Values detected 24 h prior to ablation (*n* = 100)	Values detected 24 h after ablation (*n* = 100)	Values detected 3 months after ablation (*n* = 91)	*P* ^a^	*P* ^b^
1. Verbal fluency	19 (15–22)	18 (15–22)	20 (15–24)	0.806	0.164
2. Boston naming test	15 (14–15)	15 (14–15)	15 (14–15)	0.982	0.929
3. MMSE^c^ total score	28 (27–29)	29 (27–29.75)	29 (28–30)	0.573	0.143
4. Word list learning sum	19 (16–21)	22 (19–25)	22 (19–25)	<0.001	<0.001
5. Constructional praxis	11 (8–11)	11 (9–11)	11 (10–11)	0.897	0.172
6. Word list recall	6 (5–8)	7 (5–9)	7 (6–9)	0.002	0.001
7. Word list visual recall	19 (18–20)	20 (19–20)	20 (19–20)	0.010	0.001
8. Constructional praxis recall	9 (7–11)	10 (7–12)	10 (7–12)	0.029	0.051
9. Trail making test A	43 (33–66.5)	43 (31–57)	40 (29.75–51.25)	0.369	0.042
10. Trail making test B	105 (72–181)	104 (68− 150.75)	93 (67.5–145.5)	0.371	0.165
11. Verbal fluency (s-words)	11 (9–15)	12 (8–14.75)	11 (9–16)	0.951	0.450

Presented are median values and the interquartile.

^a^ *P*-values calculated in the comparison of values detected 24 h prior to ablation and 24 h after ablation.

^b^ *P*-values calculated in the comparison of values detected 24 h after ablation and 3 months after ablation.

^c^Mini-mental state assessment.

**Table 6:** Comparison in CERAD PLUS items between patients with paroxysmal vs. persistent atrial fibrillation at Visits 1, 2, and 3

		Total group	Patients with paroxysmal	Patients with persistent	
			AF^a^	AF^a^	*P*
**Visit 1** (24 h prior ablation)		(*n* = 100)	(*n* = 50)	(*n* = 50)	
	1. Verbal fluency	19 (15–22)	19 (15–22)	18 (15–22.5)	0.856
	2. Boston naming test	15 (14–15)	15 (14–15)	15 (14–15)	0.568
	3. MMSE^b^ total score	28 (27–29)	29 (28–30)	28 (27–29)	0.589
	4. Word list learning sum	19 (16–21)	19 (17–22)	19 (16–20)	<0.001
	5. Constructional praxis	11 (8–11)	11 (9–11)	11 (7–11)	0.895
	6. Word list recall	6 (5–8)	6 (5–8)	6 (4–7)	0.512
	7. Word list visual recall	19 (18–20)	19 (18–20)	19 818–20)	0.095
	8. Constructional praxis recall	9 (7–11)	10 (7–11)	8 (7–11)	0.456
	9. Trail making test A	43 (33–66.5)	42 (33–54)	49 (35.5–73)	0.259
	10. Trail making test B	105 (72–181)	85 (70–165)	112.5 (78–213)	0.489
	11. Verbal fluency (s-words)	11 (9–15)	11 (11–15)	11 (9–14)	0.268
**Visit 2** (24 h after ablation)		(*n* = 100)	(*n* = 50)	(*n* = 50)	
	1. Verbal fluency	18 (15–22)	18.5 (14–15)	17 (14–22)	0.587
	2. Boston naming test	15 (14–15)	15 (14–15)	15 (14–15)	0.269
	3. MMSE^b^ total score	29 (27–29.75)	29 (28–29)	28 (26–29.75)	0.478
	4. Word list learning sum	22 (19–25)	22 (19.25–24.75)	22.5 (19–25.75)	<0.001
	5. Constructional praxis	11 (9–11)	11 (10–11)	11 (8–11)	0.298
	6. Word list recall	7 (5–9)	7 (5.25–8)	7 (6–9)	0.596
	7. Word list visual recall	20 (19–20)	20 (19–20)	20 (19–20)	0.458
	8. Constructional praxis recall	10 (7–12)	11 (8.25–11)	10 (7–11)	0.268
	9. Trail making test A	43 (31–57)	39 (32–52.75)	51 (31–67)	0.695
	10. Trail making test B	104 (68− 150.75)	93 (63.5–138.25)	113.5 (177.75–70)	0.493
	11. Verbal fluency (s-words)	12 (8–14.75)	12 (9–15)	11 (7–14)	0.528
**Visit 3** (3 months after ablation)		(*n* = 91)	(*n* = 48)	(*n* = 43)	
	1. Verbal fluency	20 (15–24)	21 (16.75–25)	18 (14.25–23.5)	0.235
	2. Boston naming test	15 (14–15)	15 (14.75–15)	15 (14–15)	0.412
	3. MMSE^b^ total score	29 (28–30)	29 (28–30)	28 (28–29)	0.178
	4. Word list learning sum	22 (19–25)	22 (19–25)	22 (18.25–24.5)	<0.001
	5. Constructional praxis	11 (10–11)	11 (10–11)	11 (10–11)	0.458
	6. Word list recall	7 (6–9)	7 (6–9)	7 (5.25–9)	0.359
	7. Word list visual recall	20 (19–20)	20 (19–20)	20 (19–20)	0.589
	8. Constructional praxis recall	10 (7–12)	9.5 (7–13)	10 (7–11)	0.423
	9. Trail making test A	40 (29.75–51.25)	36.5 (28.75–48.5)	45 (34–54.5)	0.216
	10. Trail making test B	93 (67.5–145.5)	83.5 (66.5–127.5)	98 (74.25–158)	0.534
	11. Verbal fluency (s-words)	11 (9–16)	13 (9.75–17)	11 (9–14)	0.238

Presented are median values and the interquartile.

^a^Atrial fibrillation.

^b^Mini-mental state assessment.

## Discussion

In this prospective observational study, involving the largest patient population ever examined for TCD detected MESs in cerebral circulation during ablation treatment exclusively using CB technology, a low MES burden was observed both in PVI and beyond. Furthermore, no impact on neurocognitive function was detected.

The presence of a bilateral acoustic bone window for the detection of MES varies widely, depending on factors such as age, gender, and ethnicity, with a reported frequency ranging from 36 to 85%.^22–24^ In our study population, which consisted of Caucasian participants, we did not find any significant differences in the presence of a unilateral vs. bilateral transtemporal acoustic bone window based on baseline characteristics. However, the detection rate of MES depends on whether a bilateral or unilateral bone window is available. In previous studies on MES detection in atrial fibrillation procedures, it was often stated that bilateral detection was attempted, but ultimately it remains unclear whether the reported MES burden derived from bilateral, unilateral, or a combination of both.^1,8,9^ This complicates the comparability of MES burden across studies. In addition, all of these studies involved small sample sizes ranging from 30 to 42 patients. In our study cohort, MES were measured bilaterally in 35 patients (36%) and unilaterally in 62 patients (64%). When indirectly comparing our data with previous atrial fibrillation ablation studies, the following can be observed: Miyazaki et al. reported a median MES burden of 522 (426–626) per patient in 40 patients with exclusively paroxysmal AF who were treated with second-generation CB.^10^ In this study, the MES burden was based on a unilateral bone window in all patients. The MES burden in our study, derived both unilaterally (median 110, IQR: 71–130) and bilaterally (median 298, IQR: 177–413), for patients with paroxysmal and persistent atrial fibrillation, is markedly lower in comparison. In contrast to the study by Miyazaki et al., we did not perform any catheter changes during the entire procedure, neither for the mapping catheter nor for the balloon catheter. We used the Achieve catheter to record intracardiac signals before, during, and after PVI. Miyazaki et al., however, measured pre- and post-pulmonary vein potentials using a Lasso catheter, which required catheter exchange via the sheath, known to be associated with a higher microembolic (gaseous) burden.^2^ Additionally, using an assist device allowed us to maintain a nearly closed system, further reducing the risk of air embolism. In ‘conventional’CB ablations, the contrast agent is administered manually via a syringe that must be refilled repeatedly, potentially allowing air to enter the system. This risk can probably be reduced by using an assist device, as in our study. All ablations in our study were performed using a fourth-generation CB balloon. It cannot be excluded that advancements in balloon technology positively influenced the MES burden observed in our study. The introduction of the new haemostatic valve on the Cryoadvance sheath by Medtronic Inc. in April 2021 did not seem to have any significant impact on the microembolic burden in our study.

Two studies comparing different ablation techniques in AF patients reported the lowest MES burden when using CB technology.^1,9^ Sauren et al. reported a mean MES count of 935 in the CB group compared with 1 404 in the irrigated RF group (*P* = 0.0019) and 3 908 in the non-irrigated RF group (*P* = 0.001) in a sample of 30 patients (*n* = 10 in each group).^9^ This study mentioned continuous bilateral insonation of the MCA ‘whenever possible’ without providing further details. In a study by Bary et al., bilateral insonation of the MCA was feasible in 35 patients (84%) and unilateral in seven patients (17%).^1^ However, they reported MES burden for the entire 42 patients; of these, 23 patients were treated with non-irrigated RF, 14 with irrigated RF, and five patients with CB technology. The total mean MES burden was significantly lower in the CB group (545, 593 ± 231) compared with the irrigated RF group (2 009, 2 336 ± 1654) (*P* = 0.007) and showed no significant difference compared with the non-irrigated RF group (602, 1 685 ± 2 255) (*P* = 0.81). Our study, which exclusively investigated the MES burden in CB ablations, supports the observations of Bary et al. and Sauren et al., particularly with our bilateral detection data, indicating that CB ablations are associated with a low MES burden.

A relevant operator dependency regarding the MES burden in bilateral recordings could not be observed in our study. As is well known, every operator has to go through a learning curve depending on the ablation technology used. Whether this influences the MES burden during the first 50 CB procedures currently remains unclear. Based on the data we collected, at least with procedural experience of 50–100 CB ablations, the MES burden no longer appears to be significantly influenced by the operator’s degree of experience.

In our study, the timely correlation of the recorded MESs with catheter manipulation allowed for the attribution of MES to their probable origin. It was observed that solid MES occurred predominantly during the freeze cycle and thawing phase, suggesting that most solid MES are related to the formation and subsequent dissolution of ice particles generated by cryoenergy. On the other hand, the solid MES recorded during transseptal puncture were more likely to be tissue fragments from the septum embolizing into the cerebral circulation, along with gas components originating from the transseptal sheath, despite prior meticulous de-airing. Likewise, it appears that extrapulmonary ablation using CB rarely induces MES, except for a few solid ones, which are likely ice particles that dissolved during the thawing phase.

While some studies report an increased microembolization rate immediately after the restoration of sinus rhythm through electrical cardioversion during PVI using RF energy, this was not observed in two previous studies that used second-generation CB technology.^25–28^ The results of our study support this latter observation, as no MES were detected at the time of cardioversion in any of the patients involved.

Several studies suggest an association between MES and silent cerebral lesions.^29–34^ These lesions were suspected to cause cognitive impairments following atrial fibrillation ablation and might increase the risk of developing dementia in the long term.^2–4,34,35^ Schwarz et al. reported a decrease in verbal memory three months after PVI in a study involving only 23 patients, 9 of whom were treated with CB and 14 with irrigated RF energy.^3^ However, the study was conducted with interrupted oral anticoagulation, with resumption of Coumadin therapy one day after PVI, aiming for an INR of 2.0–3.0. Zhang et al. observed a decline in cognitive performance in 13.7% of AF ablated patients 48 h post-procedure, which normalized after 6 months.^4^ In their study, all 190 patients were treated with irrigated RF energy. They employed a test battery consisting of nine tests as a neuropsychological assessment tool. All patients on NOAC therapy were switched to low molecular weight heparin peri-procedurally, while patients on warfarin underwent ablation with an INR value of 2.0–3.0, without bridging with heparin. The authors identified an activated clotting time of less than 300 s during the procedure and the absence of pre-procedural oral anticoagulation as risk factors for the decline in cognitive performance. Medi et al. utilized an 8-test neurocognitive test battery and reported cognitive decline in 28% of patients with ablated paroxysmal AF and 27% of patients with ablated persistent AF 48 h post-procedure.^36^ This decline persisted in 13% of patients with paroxysmal and 20% with persistent AF at the 3-month follow-up. In total, 60 patients were ablated in this study, all using irrigated RF energy. However, all ablation procedures were performed with the interruption of oral anticoagulation (warfarin) and bridging with low-molecular-weight heparin.

In the comprehensive neurocognitive testing conducted in the present study, covering a total of 11 neuropsychological domains, no significant differences in cognitive performance were observed—neither immediately 24 h after the ablation procedure nor over the course of 3 months. Interestingly, there was no measurable effect on neurocognitive ability, even in the two patients with detected pronounced gaseous microembolizations. It has been suggested that gas embolisms are less damaging to the brain than solid embolisms when occurring in the cerebral circulation.^32^ Nevertheless, it is known that gas embolisms can also lead to clinically relevant strokes. However, it seems that a critical amount of gas/air must be present for them to have a clinical impact, and this threshold was not reached in this study. The improvements in neurocognitive abilities observed in the domains of word list learning sum, word list recall, word list visual recall, constructional praxis recall, and trail making test A are all indicative of a training effect. In comparison with the studies that observed a decline in neurocognitive abilities, our study differed particularly in the anticoagulation regimen. The majority of our patients was on oral anticoagulation prior to ablation and paused it only on the morning of the procedure. Peri-interventionally, an ACT of ≥300 s was targeted, and oral anticoagulation was resumed two hours after the end of the ablation. The anticoagulation regimen implemented in our study is likely the main reason for the neurocognitive safety observed. A randomized multi-centre study involving 674 patients demonstrated that the continuous use of Apixaban or Vitamin K antagonists did not result in a reduction of cognitive abilities, despite MRI-detected acute ischaemic lesions following PVI.^37,38^ However, with all currently used ablation technologies, silent cerebral lesions can be detected on MRI after ablation treatment despite continuous oral anticoagulation, with a frequency ranging from 3 to 17%.^10,39–42^ These lesions are equally distributed across both hemispheres, with the middle cerebral artery territory being the most involved.^43^ Moreover, using high-resolution diffusion-weighted MRI, the rate of silent acute brain lesions significantly increases to up to 26% following AF ablation.^44^

Possible long-term effects, such as the promotion of dementia onset through clinically silent microembolizations during atrial fibrillation ablation, remain unclear even after this study. Several studies report that atrial fibrillation itself is an independent predictor for dementia, and its risk can be reduced by effective rhythm control, especially when using AF ablation therapy.^45–48^ Due to the continued uncertainty regarding the potential long-term neurocognitive effects of peri-procedural MES, the recommendation is to minimize their frequency as much as possible.^2^ In light of these considerations, and given that the use of cryoenergy in the described setting of our study has enabled the measurement of low microembolic signal burden during AF ablation in both paroxysmal and persistent AF patients without demonstrable compromise of neurocognitive abilities in short term follow-up, CB ablation appears to have a favourable neurocognitive safety profile.

## Limitations

The bilateral transtemporal detection rate of only 36% in the studied patient population represents a potential limitation regarding the actual microembolization rate.

## Conclusion

Doppler-detected gaseous and solid emboli are common during contemporary AF ablation. Using the CB technique, they are mainly found during contrast imaging of the pulmonary veins and the balloon freeze and thawing phase. Our observations confirm that neurocognitive abilities are not affected after AF ablation with this technique, either 24 h or 3 months after ablation. However, despite the low MES burden associated with the CB technique, more work is needed to reduce small embolic events during AF ablation.

## Data Availability

The data presented in this study are available on request from the corresponding author. The data are not publicly available due to privacy and ethical restrictions.
